# Expression of IL-17, IL-23 and Their Receptors in Minor Salivary Glands of Patients with Primary Sjögren's Syndrome

**DOI:** 10.1155/2012/187258

**Published:** 2012-01-03

**Authors:** Diana Mieliauskaite, Irena Dumalakiene, Rita Rugiene, Zygmunt Mackiewicz

**Affiliations:** Department of Experimental and Clinical Medicine, State Research Institute Center for Innovative Medicine, Zygimantu Street 9, 01102 Vilnius, Lithuania

## Abstract

The main purpose of this study was to determine the expression of interleukins-17/-23 (ILs-17/-23) and receptors of interleukins-17/-23 (IL-17R, IL-23R) in minor salivary glands (MSGs) of patients with primary Sjögren's syndrome (pSS). Expression of IL-17, IL-23 and receptors of IL-17/-23 was analyzed in MSGs from 25 patients with pSS, 25 patients with probable preclinical pSS, and 25 patients with nonautoimmune sicca syndrome by immunohistochemistry. Comparison of the expression of IL-17, IL-23 and receptors of IL-17, IL-23 in MSG of patients with pSS with probable preclinical pSS, and with nonautoimmune sicca syndrome showed significant differences between three groups. However, the expression of IL-17, IL-23 and receptors of IL-17/-23 in MSG was comparable in pSS and probable preclinical pSS patients. We did not find correlation between the expression of IL-17 and IL-23 and of IL-17R and IL-23R in patients with pSS. These results demonstrate an involvement of IL-17/-23 system in the early pSS pathogenesis.

## 1. Introduction

Primary Sjögren's syndrome (pSS) is a systemic chronic inflammatory autoimmune disorder that affects secretory organs. pSS is not only characterized by sicca syndrome but also by extraglandular manifestations that reveal the severity of this disorder. Patients with pSS also present broad spectrum analytical features (cytopenias, hypergammaglobulinemia, and cryoglobulins) and autoantibodies (antinuclear antibodies, anti-Ro/SSA, and anti-La/SSB antibodies). Thus, the spectrum of the disease ranges widely from minimal local symptoms of the eyes and oral mucosa to systemic involvement and the development of malignant lymphoma, the latter being the most worrisome complication of primary Sjögren's syndrome [1–8]. Pathophysiology of Sjögren's syndrome is not yet fully understood. Cytokines play a central role in the regulation of immunity and are often found to be deregulated in autoimmune diseases [9]. Recently, much attention has been focused on the relationship between innate responses and subsequent activation of specific adaptive immunity in an attempt to understand subsequent immune dysregulation. With the identification of CD4+ Th17 cell population, which initially challenged the long-standing Th1/Th2 paradigm, several of these important relationships between innate and adaptive immunity are now being uncovered. A subset of CD4+ memory T cells are characterized by their unique ability to secrete interleukin-17 (IL-17) family cytokines. Importantly, Th17 cells appear to be intimately involved in autoimmunity. As mature memory cells, Th17 cell survival and maintenance appear to be dependent on IL-23 [9–16].

The main purpose of this study was to determine the expression of interleukins-17/-23 and receptors of interleukins-17/-23 (IL-17R, IL-23R) focusing on autoimmune epithelitis in patients with primary Sjögren's syndrome. We set tasks to investigate a relationship between expression of IL-17 and IL-23 in patients with primary Sjögren's syndrome.

## 2. Material and Methods

### 2.1. Patients

Participants of the study were selected from patients seen at the State Research Institute Center for Innovative Medicine: 25 patients with pSS who meet the American-European criteria for primary SS, 25 patients with probable preclinical pSS who did not met fully the American-European criteria for primary SS, and 25 patients with nonautoimmune sicca syndrome. A new international consensus for Sjögren's syndrome diagnosis requires objective signs and symptoms of dryness including a characteristic appearance of a biopsy sample from a minor salivary gland or autoantibody such as anti-SSA/Ro or/and anti-SSB/La [3].

Symptoms and signs of Sjögren'syndrome are subtle and can be intermittent or nonspecific [4, 6, 17–20]. Our experience has revealed that about one-third of patients with pSS had extraglandular manifestations before the onset of sicca ocular and oral symptoms and signs including mild asymptomatic hematologic manifestations (anemia, leucopenia, and increased erythrocyte sedimentation rate). It has been estimated that these signs are present for a mean of seven years before primary Sjögren's is properly diagnosed. Patients who had mild asymptomatic hematologic abnormalities (anemia, and leucopenia, increased erythrocyte sedimentation rate), positive anti-SSA/Ro or/and anti-SSB/La, and negative serology for hepatitis C, EBV, cytomegalovirus, and human immunodeficiency virus were included in the probable preclinical pSS group. All patients underwent an extensive medical examination. Following the initial evaluation by a rheumatologist, each patient was referred to Sjögren's syndrome specialists for a review of his/her medical history, an oral and an ocular examination, Schirmer's *I* test, unstimulated salivary flow rate, and a labial gland biopsy. Also, all patients underwent extensive serologic evaluations which included test for the presence of rheumatoid factor (RF), antinuclear antibodies (ANA), anticentromere antibodies, anti-double-stranded DNA, anti-Scl-70, anti-Sm, anti-SSA/Ro, and anti-SSB/La (Table 1). Patients of all groups had negative serology for hepatitis C, EBV, cytomegalovirus, and human immunodeficiency virus.

Informed and written consent was obtained from all patients who participated in this study. The study has been approved by the Lithuanian Bioethics Committee (2009-06-03, No.: 158200-6-061-15).

### 2.2. Labial Salivary Gland Biopsy

Labial salivary gland biopsies were performed on patients by the oral medicine specialist. A local anesthetic was injected into the lower lip followed by a small incision to the right or left of the lip midline. Five or six minor salivary gland lobules were harvested and placed into formalin fixative. Standard paraffin preparations were prepared, sectioned, and stained with hematoxylin and eosin. The slides were examined for the presence of lymphocytic infiltrates by 2 board-certified pathologists.

### 2.3. Immunohistochemical Staining

Biopsy specimens from the lips of patients were placed in 10% phosphate-buffered formalin for 24 hours. Fixed tissues were embedded in paraffin and sectioned at 4 *μ*m thick sections with *Microm *microtome. 4 *μ*m tissue sections were deparaffinized by immersion in xylene followed by dehydratation in ethanol. Antigen retrieval was performed in 0.01 M sodium citrate buffer, pH 6.0, at +98°C for half an hour in *Milestone *histoprocessor or with 0.05% trypsin solution (pH 7.8) for 20 minutes at 37°C., followed by cooling down at +22°C for 30 min. Endogenous peroxidase was blocked with 0.3% H_2_O_2_ in methanol for 30 minutes at +22°C. Following a 5-minute wash with PBS, sections were incubated at +22°C for 30 minutes with primary antibody at the room temperature.

Primary antibodies were used: rabbit anti-human IL-17 (H-132) polyclonal antibody, 2 *μ*g/mL (Santa Cruz Biotechnology, Inc, Santa Cruz, CA, USA); goat anti-human IL-17 RD/SEF receptor polyclonal IgG, 4 *μ*g/mL (R&D Systems, Minneapolis, MN, USA); mouse anti-human IL-23 (p19) monoclonal IgG1, *κ*, 4 *μ*g/mL (BioLegend, San Diego, CA, USA); goat anti-human IL-23 receptor polyclonal antibody, 4 *μ*g/mL (Capralogics, Inc. Hardwick, MA, USA).

Incubation with appropriate biotinylated secondary antibodies: rabbit anti-goat, horse anti-mouse, or goat anti-rabbit, all from the VECTASTAIN ABC kit, 1 : 200 (Vector Laboratories, Burlingame, CA, USA), was performed at +22°C for half an hour and was used following manufacturer's instructions. All incubations were performed in humid atmosphere. The staining was developed by using diaminobenzidine substrate (Vector Laboratories, Burlingame, CA, USA), and counterstaining was performed with hematoxylin (Merck, Darmstadt, Germany), followed by mounting in HistoGel (Lab. Storage Systems Inc., St. Peters, MO, USA). Slides were rinsed in PBS 2 × 5 minutes after each step.

### 2.4. Evaluation of the Results

Semiquantitative microscopic assessment of immunohistochemical staining was performed in light microscope *Olympus BX51 *under ×400 (high-power fields) scoring 5 random fields in 10 random similarly stained slides using four grades: “0” = no immunoreactivity; “1” = low immunoreactivity; “2” = moderate immunoreactivity; “3” = high immunoreactivity. In every slide were separately scored: (1) acini; (2) glandular ducts; (3) intraglandular interstitium; (4) blood vessels; (5) infiltrating inflammatory cells. The immunoreactivity grades of IL-17, IL-23, IL-17R, and IL-23R in the separate localizations sum is a total expression of mentioned interleukins and their receptors. Theoretically, maximum score could reach the value of 15. Practically, such a value never occurred in this study. Two histologists evaluated the results of histological and immunohistochemical staining independently using this predefined and a very simple scoring system leading to very similar readouts and followed by a consensus session.

### 2.5. Statistical Analysis

All values presented are the mean ± SD. Statistical differences were analyzed with the Mann-Whitney, Kruskal-Wallis tests using standard program SPSS 19.0. *P* values less than 0.05 were considered significant.

## 3. Results

**Figure 1:**
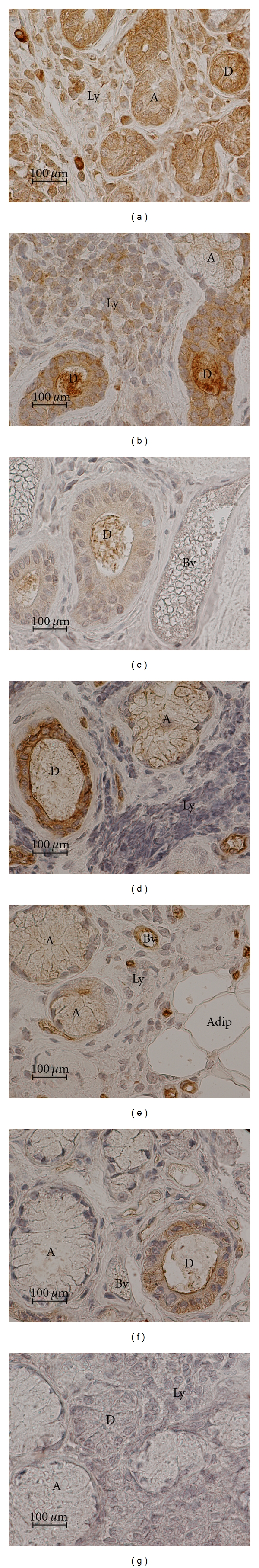
Expression of IL-17 and IL-17R in minor salivary glands of patients with pSS, probable preclinical pSS, and nonautoimmune sicca syndrome. IL-17: (a) pSS, (b) probable preclinical pSS, (c) nonautoimmune sicca syndrome. IL-17R: (d) pSS, (e) probable preclinical pSS, (f) nonautoimmune sicca syndrome, (g) negative staining control. Counterstained with hematoxylin. Original magnification ×400. Abbreviations: A: acinus, Adip-adipocytes, Bv: blood vessel, D: ductus, and Ly: lymphocyte infiltration.

**Figure 2:**
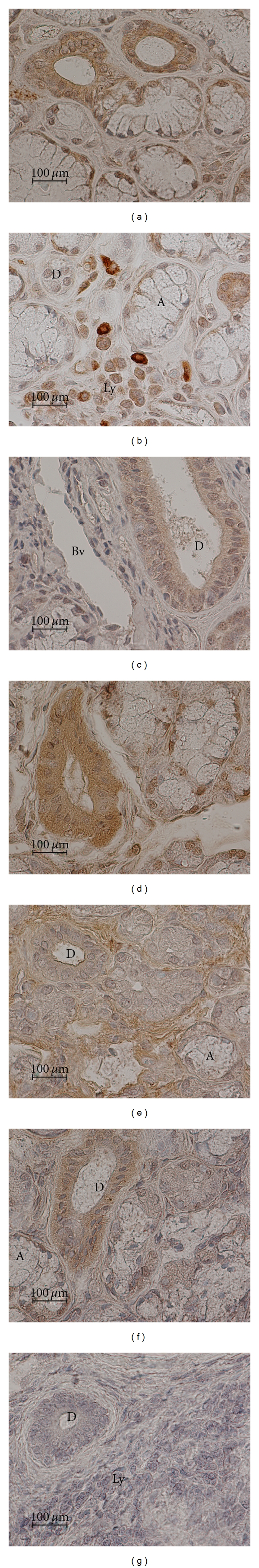
Expression of IL-23 and IL-23R in minor salivary glands of patients with pSS, probable preclinical pSS and nonautoimmune sicca syndrome. IL-23: (a) pSS, (b) probable preclinical pSS, (c) nonautoimmune sicca syndrome. IL-23R: (d) pSS, (e) probable preclinical pSS, (f) nonautoimmune sicca syndrome, (g) negative staining control. Counterstained with hematoxylin. Original magnification ×400. Abbreviations: A: acinus, Bv: blood vessel, D: ductus, and Ly: lymphocyte infiltration.

IL-17, IL-23, IL-17R, and IL-23R were detected in glandular ducts (d), acini (a), blood vessels (bl v), intraglandular interstitium (i), infiltrating inflammatory cells (ly) of minor salivary glands (MSGs) of patients with pSS (*n* = 25), probable preclinical pSS (*n* = 25), and nonautoimmune sicca syndrome (*n* = 25). Representative photomicrographs of the expression patterns in patients with pSS, probable preclinical pSS, and nonautoimmune sicca syndrome are shown in figures (Figures 1 and 2).

Assessment of the total expression of IL-17, IL-23 and receptors of IL-17, IL-23 in MSGs of patients with pSS, probable preclinical pSS, and nonautoimmune sicca syndrome and comparison of the total expression of IL-17, IL-23 and receptors of IL-17, IL-23 between three groups showed significant differences (*P* < 0.05) (Tables 2 and 3). The mean total expression of IL-17, IL-23 and receptors of IL-17, IL-23 was increased in MSGs of pSS patients compared with nonautoimmune sicca syndrome patients (*P* < 0.05). However, the total expression of IL-17, IL-23 and receptors of IL-17, IL-23 in MSGs of pSS was comparable to probable preclinical pSS patients. Comparison of the total expression of IL-17, IL-23, and IL-17R between probable preclinical pSS and nonautoimmune sicca syndrome patients showed significant differences. Furthermore, the highest expression of IL-17, IL-23 was revealed in glandular ducts and inflammatory cells of patients with pSS and probable preclinical pSS. Expression of IL-17R in glandular ducts was increased in MSGs of pSS (*P* < 0.05) and was comparable in the glandular ducts of patients with probable preclinical pSS and nonautoimmune sicca syndrome.

The total expression of IL-23 was increased in MGS of pSS patients compared with nonautoimmune sicca syndrome patients. We found the highest expression of IL-23 in glandular ducts and acini of patients with pSS and probable preclinical pSS. The IL-23 expression in blood vessels, intraglandular interstitium, and infiltrating inflammatory cells of minor salivary glands was comparable in three groups. Our study revealed that the total expression of IL-23R was significantly higher compared with the total expression of IL-23 in MSGs of patients with probable preclinical pSS. The total expression of IL-23R and of IL-23R in glandular ducts, acini, intraglandular interstitium, and infiltrating inflammatory cells was comparable in pSS and probable preclinical pSS patients. Furthermore, we found that the total expression of IL-23R and of IL-23R in glandular ducts, acini, intraglandular interstitium, and infiltrating inflammatory cells was significantly higher in pSS and probable preclinical pSS patients compared with nonautoimmune sicca syndrome patients.

Expression of IL-17/IL-23 and IL-17R/IL-23R significantly correlated in minor salivary glands of patients with probable preclinical pSS (accordingly, *r* = 0.683, *P* < 0.001 and *r* = 0.406, *P* = 0.044) and nonautoimmune sicca syndrome patients (accordingly, *r* = 0.740, *P* < 0.001 and *r* = 0.506, *P* = 0.010). We did not find correlation between expression of IL-17R and IL-23R in patients with pSS (*r* = 0.181; *P* = 0.386).

## 4. Discussion

In this study, we sought to determine the expression of interleukins-17/-23 (IL-17/-23) and receptors of interleukins-17/-23 (IL-17R, IL-23R) in minor salivary glands (MSGs) of patients with primary Sjögren's syndrome (pSS), with probable preclinical pSS, and with nonautoimmune sicca syndrome. Patients who had mild asymptomatic hematologic abnormalities (anemia, leucopenia, and increased erythrocyte sedimentation rate), positive anti-SSA/Ro or/and anti-SSB/La, and negative serology for hepatitis C, EBV, cytomegalovirus, and human immunodeficiency virus were included in the probable preclinical pSS group. Among the antinuclear autoantigens targets, the ribonucleoprotein particles (Ro/SSA and La/SSB) have a prominent role in pSS diagnosis and systemic activity [6, 17].

Cytokine-mediated immunity plays a substantial role in the pathogenesis of various autoimmune diseases including primary Sjögren's syndrome. Dysregulation of the cytokine network contributes to both systemic and exocrine manifestations of SS [9]. Recently some studies have added primary Sjögren's syndrome to the rapidly expanding list of autoimmune diseases (e.g., multiple sclerosis, systemic lupus erythematosus, rheumatoid arthritis, and psoriasis) in which cytokines IL-17 and IL-23 are now implicated [12, 21–27].

Besides being produced by Th17 cells, IL-17A is also produced by a variety of cell types, including NK cells, neutrophils, and eosinophils. Thus, IL-17 is an effector cytokine that is produced by cells of both the innate and the adaptive immune systems, suggesting a bridging function of this type of immunity between innate and adaptive immune responses. IL-17A has proinflammatory properties and act on broad range of cell types to induce expression of cytokines, chemokines, and metalloproteinases. IL-17 was reported to be a factor that contributes to the formation of germinal centers (GCs) of lymph follicles retaining B cells within GCs an enhancing through modulation of chemokine activity. The IL-17 receptors constitute a distinct family of cytokine receptors. IL-17RA and IL-17RC are the receptors for IL-17A and IL-17F. IL-17RA not only conveys proinflammatory IL-17 effects, but also contributes in IL-25 signaling. IL-17RA binds IL-17A with higher affinity compared with IL-17F. IL-17RA is not only highly expressed on hematopoietic cells, but also at lower levels on endothelial cells, epithelial cells, osteoblasts, and fibroblasts [10, 15].

Recent studies revealed that IL-23, not IL-12, is regarded as a crucial cytokine for the pathogenesis of autoimmune diseases. IL-23 plays a key role in the development of pathogenetic Th17 cells that produce the cytokine IL-17 and finally contribute to hyperproduction of IL-17 and other cytokines. IL-23 is expressed predominantly by activated dendritic and phagocytic cells. IL-12 and IL-23 share a common p40 subunit binding to common IL-12 receptor (IL-23R). IL-23R is expressed on activated/memory T cells, T-cell clones, and natural killer cell lines in human. IL-23R has now been proposed as a common genetic marker for a variety of autoimmune diseases [28].

Comparison of the total expression of IL-17 and IL-17R in minor salivary glands between three groups showed significant differences. However, the total expression of IL-17 and IL-17R in MSGs of pSS was comparable to probable preclinical pSS patients. The highest expression of IL-17 was revealed in glandular ducts and inflammatory cells of patients with pSS and probable preclinical pSS. Resent study also revealed that the staining of the IL-17 and IL-23 appeared localized to lymphocytic infiltrates and ductal cells, with less staining occurring in acini. Another resent study showed that ageing induces specific changes in lacrimal-keratoconjunctivitis in CD25KO mice, with a mix of Th1 and Th17 cytokines, and that the peak severity of corneal epithelial disease corresponded to the peak of IL-17 expression [29].

The expression of IL-17R in glandular ducts was significantly higher in pSS patients compared with probable preclinical pSS patients, but the expression of IL-17R in acini of pSS patients was comparable to probable preclinical pSS patients. These results suggest that autoimmune inflammation may appear in salivary glands before sicca symptoms and signs in patients with probable pSS.

The total expression IL-23 and IL-23R in minor salivary glands of pSS was comparable to probable preclinical pSS patients. In contrast to our results, before-mentioned study revealed that IL-17 expression appeared stronger and more widely distributed than IL-23 expression in pSS patients [12]. We suppose that this contrast might be explained by different sizes of study groups. We found the highest expression of IL-23 in glandular ducts and acini of patients with pSS and probable preclinical pSS. Our study revealed that the total expression of IL-23R was significantly higher compared with the total expression of IL-23 in MSGs of patients with probable preclinical pSS. The total expression of IL-23R and expression of IL-23R in glandular ducts, acini, intraglandular interstitium, and infiltrating inflammatory cells was comparable in pSS and probable preclinical pSS patients. These results may demonstrate an influential role of IL-23R in the onset of autoimmune epithelitis. Future investigations need to take these data into consideration in designing studies to examine effects of IL-23R. Furthermore, IL-23R has now been proposed as a common genetic marker for a variety of autoimmune diseases [28].

The expression of IL-17/IL-23 and IL-17R/IL-23R significantly correlated in minor salivary glands of patients with probable preclinical pSS and nonautoimmune sicca syndrome patients. We did not find correlation between expression of IL-17R and IL-23R in patients with pSS. These data reflect that cytokines networks may contribute to the pathogenesis of SS in various and separate ways.

We speculate that the particular expression of interleukins-17/-23 and their receptors may reflect ongoing autoimmune inflammation in various target organs and suggest a role for these interleukins and their receptors in the early SS stage.

## 5. Conclusion

These results demonstrate an involvement of interleukin-17/-23 in the early Sjögren's syndrome pathogenesis.

## Figures and Tables

**Table 1:** Clinical and serologic characteristics of the patients.

Variable	pSS (*n* = 25)	Probable preclinical pSS (*n* = 25)	Nonautoimmune sicca syndrome (*n* = 25)
Age, mean (s.d)	59.6 (11.9)	58.4 (13.4)	63.1 (10.1)
Ocular symptoms, *n* (%)	25 (100)	0 (0)	25 (100)
Oral symptoms, *n* (%)	25 (100)	0 (0)	25 (100)
Schirmer *I* test, ≤5 mm/5 min., *n* (%)	25 (100)	0 (0)	25 (100)
Unstimulated salivary flow rate, ≤1.5 mL/15 min., *n* (%)	25 (100)	0 (0)	25 (100)
Salivary secretion, mean (s.d)	1.0 (0.22)	2.1 (0.4)	1.4 (0.17)
Histopathology, focus score ≥1, *n* (%)	25 (100)	20 (80)	0 (0)
Salivary gland enlargement, *n* (%)	5 (20)	0 (0)	0 (0)
Arthralgias, *n* (%)	18 (72)	15 (60)	14 (56)
Raynaud phenomenon, *n* (%)	3 (12)	1 (4)	0 (0)
Cutaneous vasculitis, *n* (%)	1 (4)	0 (0)	0 (0)
Pulmonary involvement, *n* (%)	3 (12)	0 (0)	0 (0)
Renal involvement, *n* (%)	0 (0)	0 (0)	0 (0)
Fatigue, *n* (%)	17 (68)	19 (76)	0 (0)
ESR, ↑, *n* (%)	7 (28)	12 (48)	0 (0)
Leucopenia, *n* (%)	6 (24)	8 (32)	0 (0)
Anemia, *n* (%)	8 (32)	6 (24)	0 (0)
RF, >15 kU/I, *n* (%)	25 (100)	25 (100)	25 (100)
ANA, >1 : 40, *n* (%)	25 (100)	25 (100)	25 (100)
ACA, negative, *n* (%)	25 (100)	25 (100)	25 (100)
Anti-dsDNA, negative, *n* (%)	25 (100)	25 (100)	25 (100)
Anti-Scl-70, negative, *n* (%)	25 (100)	25 (100)	25 (100)
Anti-Sm, negative, *n* (%)	25 (100)	25 (100)	25 (100)
Anti-Ro/SSA, *n* (%)	21(84)	23 (92)	0 (0)
Anti-La/SSB, *n* (%)	0 (0)	0 (0)	0 (0)
Both anti-Ro/SSA and anti-La/SSB, *n* (%)	4 (16)	2 (8)	0 (0)

**Table 2:** Expression (grades) of IL-17 and IL-17R in minor salivary glands of patients with primary Sjögren's syndrome (pSS), with probable preclinical primary Sjögren's syndrome (probable preclinical pSS), and with nonautoimmune sicca syndrome: ducti (d), acini (a), blood vessels (bl v), interstitium (i), lymphocytes (ly)] [mean ± SD, median (*M*)].

Variable	pSS		Probable preclinical pSS		Nonautoimmune sicca syndrome	
	Mean ± SD	*M*	Mean ± SD	*M*	Mean ± SD	*M*
IL-17	4.6 ± 1.38^∗,2,3^	5.0	4.32 ± 1.31	4.0	3.8 ± 1.15	3.0
IL-17 a	0.88 ± 0.33^∗,2,3^	1.0	0.96 ± 0.54	1.0	0.56 ± 0.51	1.0
IL-17 d	1.68 ± 0.48^∗,2^	2.0	1.44 ± 0.51	1.0	1.20 ± 0.41	1.0
IL-17 i	0.60 ± 0.50^∗,1,2^	1.0	0.16 ± 0.37	0.0	0.12 ± 0.33	0.0
IL-17 bl v	0.32 ± 0.56^∗,1,3^	0.0	0.60 ± 0.50	1.0	0.20 ± 0.41	0.0
IL-17 ly	1.16 ± 0.37^2,3^	1.0	1.20 ± 0.41	1.0	1.00 ± 0.00	1.0
IL-17R	4.68 ± 1.84^∗,2,3^	5.0	4.44 ± 1.89	4.0	3.08 ± 1.68	3.0
IL-17R a	0.88 ± 0.33^∗,2,3^	1.0	0.96 ± 0.45	1.0	0.56 ± 0.51	1.0
IL-17R d	1.68 ± 0.48^∗,1,2^	2.0	1.32 ± 0.48	1.0	1.20 ± 0.41	1.0
IL-17R i	0.24 ± 0.44	0.0	0.44 ± 0.51	0.0	0.20 ± 0.41	0.0
IL-17R bl v	1.04 ± 0.79^∗,2,3^	1.0	0.84 ± 0.55	1.0	0.48 ± 0.51	0.0
IL-17R ly	0.84 ± 0.37	1.0	0.88 ± 0.44	1.0	0.64 ± 0.49	1.0

*P < 0.05, comparison between all groups (Kruskal-Wallis test);

*¹P* < 0.05, comparison between patients with pSS and probable preclinical pSS (Mann-Whitney *U* test);

²*P* < 0.05, comparison between patients with pSS and nonautoimmune sicca syndrome (Mann-Whitney *U* test);

³*P* < 0.05, comparison between patients with probable preclinical pSS and nonautoimmune sicca syndrome (Mann-Whitney *U* test).

**Table 3:** Expression (grades) IL-23 and IL-23R in minor salivary glands of patients with primary Sjögren's syndrome (pSS), with probable preclinical primary Sjögren's syndrome (probable preclinical pSS), and with nonautoimmune sicca syndrome: ducti (d), acini (a), blood vessels (bl v), interstitium (i), lymphocytes (ly)], [mean ± SD, median (*M*)].

Variable	pSS		Probable preclinical pSS		Nonautoimmune sicca syndrome	
	Mean ± SD	*M*	Mean ± SD	*M*	Mean ± SD	*M*
IL-23	4.08 ± 1.75^∗,2^	4.0	3.32 ± 1.35	4.0	2.68 ± 1.68	3.0
IL-23 a	0.96 ± 0.54^∗,2,3^	1.0	0.84 ± 0.47	1.0	0.52 ± 0.51	1.0
IL-23 d	1.64 ± 0.49^∗,2,3^	2.0	1.44 ± 0.51	1.0	1.08 ± 0.49	1.0
IL-23 i	0.28 ± 0.46	0.0	0.2 ± 0.41	0.0	0.32 ± 0.48	0.0
IL-23 bl v	0.24 ± 0.44	0.0	0.16 ± 0.37	0.0	0.16 ± 0.37	0.0
IL-23 ly	0.88 ± 0.60	1.0	0.68 ± 0.48	1.0	0.64 ± 0.57	1.0
IL-23R	4.28 ± 1.97^∗,2,3^	4.0	4.52 ± 1.96	5.0	2.76 ± 1.79	3.0
IL-23R a	0.96 ± 0.54^∗,2,3^	1.0	0.80 ± 0.41	1.0	0.52 ± 0.51	1.0
IL-23R d	1.52 ± 0.51^∗,2,3^	2.0	1.36 ± 0.49	1.0	1.0 ± 0.41	1.0
IL-23R i	0.48 ± 0.51^∗,3^	0.0	0.76 ± 0.52	1.0	0.48 ± 0.51	0.0
IL-23R bl v	0.44 ± 0.51	0.0	0.72 ± 0.54	1.0	0.20 ± 0.41	0.0
IL-23R ly	0.88 ± 0.44^∗,2,3^	1.0	0.88 ± 0.44	1.0	0.56 ± 0.51	1.0

*P < 0,05, comparison between all groups (Kruskal-Wallis test);

*¹P* < 0,05, comparison between patients with pSS and probable preclinical pSS (Mann-Whitney *U* test);

²*P* < 0,05, comparison between patients with pSS and nonautoimmune sicca syndrome (Mann-Whitney *U* test);

³*P* < 0,05, comparison between patients with probable preclinical pSS and nonautoimmune sicca syndrome (Mann-Whitney *U* test).
